# Mid-Term Outcomes of Arthroscopic Treatment in Patients with a Stiff Elbow

**DOI:** 10.7759/cureus.2630

**Published:** 2018-05-15

**Authors:** Reşit Sevimli, Okan Aslantürk, Emre Ergen, Kadir Ertem

**Affiliations:** 1 Department of Orthopaedics and Traumatology, Inonu University School of Medicine, Malatya, TUR

**Keywords:** elbow, joint diseases, arthroscopy, joint capsule release

## Abstract

Introduction

Loss of function and pain are the main complaints at the time of hospital admission for patients with a stiff elbow. In this study, we present mid-term radiological and functional results for the use of the arthroscopic release technique in patients admitted to the outpatient clinic with a stiff elbow.

Methods

A total of 22 patients (six females, 16 males; mean age: 36 years, range: 18 to 56 years) who underwent an arthroscopic intervention for traumatic or non-traumatic stiff elbow and arthrosis between January 2005 and November 2015 were included in the study. All patients started elbow movement after the first day following surgery. Pre- and postoperative radiological evaluations of patients were carried out, in addition to functional evaluation to measure the range of motion of the elbow joint and the Disabilities of the Arm, Shoulder and Hand (DASH) scores before and after surgery.

Results

The mean follow-up was 28.4 (range: 21 to 118) months. The mean preoperative flexion-extension arc of the patients was 89° (range: 0° to 115°), and the mean flexion-extension arc increased to 103.5° (range: 52° to 128°) at the final follow-up visit (p < 0.05). The mean preoperative DASH score was 42.17 (range: 33 to 81), decreasing to 30.35 (range: 9.7 to 41.3) postoperatively (p<0.05). In the final visit, none of the patients were found to require additional surgical interventions for the elbow.

Conclusions

Arthroscopic release can be considered a safe and effective option to obtain range of motion in joints in post-traumatic stiff elbow cases.

## Introduction

Elbow stiffness due to traumatic or non-traumatic events is a common and difficult-to-treat problem that affects the daily activities and the quality of life of sufferers [1]. The etiology of post-traumatic stiffness is multi-factorial, including joint degeneration, heterotopic ossification, post-fracture joint dislocation, and soft tissue stiffness [2]. An arthroscopic intervention provides a better field of vision around the elbow joint and allows capsule release, debridement, and fragment excision during operation, while also reducing surgical trauma and initiating early rehabilitation [3-4]. Given the benefits of arthroscopic interventions, in the present study, we seek to evaluate the mid-term radiological and functional results of the arthroscopic release technique and free-fragment excision in patients admitted to our outpatient clinic with a stiff elbow.

## Materials and methods

Patients who underwent an arthroscopic intervention for elbow stiffness (eight non-traumatic, 14 post-traumatic) between January 2005 and November 2015 were included in the study, which was approved by the Ethics Committee of the Scientific Research and Publications Board of Inonu University (Approval No. 2017/2-1). Inclusion criteria were as follows: availability of follow-up data for the patient for at least six months after the operation, absence of mental impairment or disability, and absence of secondary hardness (primary osteoarthrosis, burns, or ossifying myositis). The dominant right elbow was affected in 11 patients, and the non-dominant left elbow in 11 patients. In the etiology, 14 of the injuries were post-traumatic, and eight were non-traumatic (epicondylitis, loose body, juvenile rheumatoid arthritis (JRA) arthrosis). In 14 patients with post-traumatic arthrosis, stiff elbow developed in eight after a fall from a height, four had in-vehicle traffic accidents, and two had minor trauma fractures. In addition to conventional two-dimensional x-ray imaging, all patients were evaluated with a three-dimensional computed tomography (CT) scan, leading to an operation decision. Radiological images of the patients revealed loose body-like fragments in eight patients and arthrosis and osteophytes in 12 patients.

Surgical technique

All surgeries were performed by the same surgeon (KE). The patients were positioned in the prone position under general anesthesia and a pneumatic tourniquet was applied to the upper end of the bracket. The third upper-middle part of the arm was placed on an arm support locked to the operation table. A 90⁰ elbow flexion position was achieved, and full elbow flexion and extension movements were allowed. The soft spot on the elbow (at the center of the olecranon-radial head-lateral epicondylitis trigon), and the proximal medial and proximal lateral were marked. At the lateral part of the elbow joint, an 18G spinal needle was inserted into the joint from the soft spot between the olecranon, radial head, and lateral epicondyle of the elbow, and the joint was inflated with 25–30 cc of saline. Then, 2 cm proximal of the medial epicondyle was marked. After a blunt dissection of the subcutaneous tissue with the help of hemostats, entry was made from the anterior part of the medial intermuscular septum and tissues were removed from the front capsule. The skin incision was made with a scalpel (number 11), and a blunt trocar was advanced through this gap. The capsule was passed and the joint was entered. Then, a standard 4.5 mm arthroscope with a 30° angulation was placed in the lateral compartment of the elbow, and the radiocapitellar joint was reached. A 30 mmHg pressurized irrigation pump was used during the surgery. In the joint, from the lateral to medial, capsule, the lateral border of capitellum, capitellum, radial head, trochlea humeri, coronoid process, medial compartment of the joint, and the medial groove were observed, respectively. These portals were used for anterior capsule release (along the proximal humeral attachment), for the removal of the coronoid process osteophytes and any anterior compartment loose bodies, and for the debriding of the damaged radial head. Posterior compartment procedures were performed after the anterior compartment. For visualization and for working in the posterior compartment, the direct posterior and posterolateral portals were used, respectively. An arthroscope was introduced through the direct posterior portal, located 3 cm proximal to the olecranon tip, and the posterolateral portal was established approximately 3 cm proximal to the olecranon tip, close to the lateral margin of the triceps. Using these portals, a posterior capsulotomy was performed in cases with decreased flexion, and an anterior capsulotomy in patients with restricted extension. Olecranon osteophytectomy and olecranon fossa deepening were performed in patients with kissing osteophytes, which restricted the range of motion (ROM). A similar indication was used for coronoid fossa deepening and coronoid process osteophytectomy processes. No resection of the medial collateral ligament to achieve greater flexion was performed in any of the patients. Finally, a Hemovac drain (Zimmer Biomet, Warsaw, Poland) was applied to the joint and the portals were sutured. The elbow was wrapped with compressive bandages (Table *1*).

**Table 1:** Arthroscopic procedures

Procedure	No	%
Loose body removal	8	36
Anterior capsular release	14	63
Posterior capsular release	14	63
Olecranon osteophytectomy	17	77
Olecranon fossa deepening	18	81 (77.1%)
Coronoid fossa osteophytectomy	16	72
Coronoid fossa deepening	14	63

Postoperative care

Appropriate doses of intravenous antibiotics and analgesics (tenoxicam) were administrated to all patients in the first 24 hours. The Hemovac drain was removed the next day, and after an x-ray graphic and controlled CT, early active and passive ROM exercises were begun in the physical therapy and rehabilitation unit.

Statistical analysis

The MedCalc statistical software (MedCalc Software Ostend, Belgium) was used for the statistical analysis, with descriptive data expressed as a median. A student's T-test was used to compare the ROM and Disabilities of the Arm, Shoulder and Hand (DASH) scores before and after surgery. A p-value less than 0.05 was considered statistically significant.

## Results

Of the total 22 patients, six were female and 16 were male, and the mean age was 36 (range: 18 to 56) years. The mean patient follow-up period was 28.4 months (range: 21 to 118). In the post-traumatic group, the preoperative flexion-extension arc was 81.5° (SD ± 37.549), increasing to 93° (SD ± 49.589) after surgery (p < 0.05), while the mean preoperative DASH score was 46.87 (SD ± 23.587), increasing to 38.46 (SD ±  24.687) postoperatively (p < 0.05) (Table *2*).

**Table 2:** Preoperative and postoperative ROM and DASH scores ROM: range of motion; DASH: Disabilities of the Arm, Shoulder and Hand  scores; SD: standard deviation

	Preop	Postop	p	CI
ROM	89.14 (SD ± 36.855)	103.5 (SD ± 45.492)	0.001	95%
DASH	42.17 (SD ± 24.825)	30.35(SD ± 26.198)	0.008	95%

In the non-traumatic group, the preoperative flexion-extension arc was 102.5° (SD ± 33.7), increasing to 121.88° (SD ± 32.176) after surgery (p < 0.05), while the mean preoperative DASH score was 33.95 (SD ± 26.347), increasing to 16.16 (SD ± 23.777) postoperatively (p < 0.05). The mean preoperative flexion-extension arc was 89.14° (SD ± 36.855), and flexion contracture was 64°. The mean flexion-extension arc had increased to 103.5° (SD ± 45.492°) at the final follow-up (p<0.05). The mean preoperative DASH score was 42.17 (SD ± 24.825), increasing to 30.35 (SD ± 26.198) postoperatively (p < 0.05). A statistically significant difference was identified only in the postoperative DASH scores between the post-traumatic and non-traumatic groups. No complications were observed in the early postoperative period (Table *3*).

**Table 3:** Traumatic and non-traumatic groups in terms of only postop DASH values DASH: Disabilities of the Arm, Shoulder and Hand  scores

	N	Preop ROM	Postop ROM	Preop DASH	Postop DASH
Traumatic	14	81.5 (SD ± 37.549)	93 (SD ± 49.589)	46.87 (SD ± 23.587)	38.46 (SD ± 24.687)
Non-traumatic	8	102.5 (SD ± 33.7)	121.88 (SD ± 32.176)	33.95 (SD ± 26.347)	16.16 (SD ± 23.777)

## Discussion

A number of open surgical procedures have been used to open the contracture in patients suffering from stiff elbow in the literature [5-6]. Many procedures have resulted in additional injuries to the soft tissue during surgery, or have led to difficulties in early physiotherapy and an increased risk of recurrence of contractility [7-9]. Open techniques may restrict the extensive examination of all structures that may cause elbow stiffness [10-12]. In this study, involving cases of both post-traumatic and non-traumatic stiff elbow, we achieved a statistically significant improvement in the flexion-extension arc and in flexion contracture after arthroscopic release, where standard physical therapy protocols were applied. Morrey [12] reported that in 26 patients (25 of which were followed-up), the mean preoperative total motion arc was 30° (63° to 93° flexion), increasing to 96° (30 to 126°) during the postoperative follow-up examinations. Like the Morrey study [12], Larson et al. reported a mean preoperative flexion-extension arc increase from 51° to 97° postoperatively in 38 patients with elbow contracture who were treated with interposition arthroplasty with an Achilles tendon allograft [13]. In all of the patients treated for the stiff elbow in the present study (both post-traumatic and non-traumatic), we achieved significant improvement in the mean flexion-extension arc and mean flexion contracture, consistent with the clinical presentation.

Hard elbow disease is difficult to treat, and patient satisfaction is low. Regaining ROM improves functional outcomes in most cases.  Elbow arthroscopy is a minimally invasive method, and while its learning time is relatively long, it permits a better visualization and manipulation of more fields than in open techniques [14]. In addition, better outcomes can be expected from elbow arthroscopy due to the lower aggressiveness to soft tissues and the more cosmetic character of the procedure [14]. Arthroscopic release in stiff elbow patients follows the same steps as an open release, with the only difference being the mode of insertion. The most important result obtained from this study is that arthroscopic release in stiff elbow cases can be considered an effective and reliable treatment method, regardless of whether the complaint is post-traumatic or non-traumatic. There are many studies supporting the success of arthroscopic release in the literature [15], and it was found to be successful in all of our patients (both post-traumatic and non-traumatic) when secondary reasons, such as myositis ossificans or burns, were excluded.

In addition to the advantages of arthroscopic release, such as the wider visualization of the joint, the smaller incision and associated post-traumatic scar problems, and the faster starting of postoperative rehabilitation programs and the shorter hospitalization periods in these cases, there are also a number of potential complications [16]. These include the possibility of neurovascular injury during the opening of the portals, the inability to intervene in post-traumatic cases in which the joint space is quite narrow and there is limited area of operation within the joint, as well as such secondary reasons such as heterotopic ossification [17]. These can be put forward as the limitations of the arthroscopic release technique. Ensuring that the elbow arthroscopy is carried out by a surgeon who has completed the learning curve reduces the risk of complications, as highlighted by WuHong, et al. [18].

Arthroscopic findings in the joint during an elbow arthroscopy for stiff elbow may vary according to etiology [19]. In post-traumatic cases, the joint space is narrower, and the capsule is more fibrotic and more continuous, while in degenerative cases, the space of the joint is wider, the capsule is softer, and the continuity is lower [20]. During this procedure, which should be performed by a surgeon experienced in arthroscopy, different portals or different techniques may be required to address the underlying pathology of stiffness; for example, anterior and posterior capsule release, deepening of the olecranon fossa, or excision of osteophytes in the olecranon or coronoid fossa may be required. Anterior capsulectomies and deepening the olecranon fossa through the excision of the osteophytes in the olecranon fossa may increase elbow extension, while the excision of osteophytes in the coronoid fossa or anterior distal humerus with posterior capsulectomy may increase elbow flexion [21]. Attention should be paid to the brachialis muscle due to the risk of brachial artery injury when making an anterior capsulectomy. Avoiding muscle damage may also reduce the risk of heterotopic ossification [21]. Brachial muscles have been found to be quite thin due to atrophy in stiff elbow cases, as reported by Kelly et al. [22].

Open release operations usually have good results, but in patients with stiff elbow, it is necessary to initiate preventive and enhancing rehabilitation programs as soon as possible after surgery, and wider incisions and more soft tissue trauma than arthroscopic release delay the initiation of rehabilitation programs. There have been a number of studies in the literature, including cases operated on by more than one surgeon. Reddy et al. [23] based their study on data from more than one clinic and from different surgeons in long-term clinical trials; however, this may result in the use of different surgical and rehabilitation protocols. In our study, all of the patients were operated on by the same surgeon and the same physiotherapy protocol was performed after surgery.

The number of patients in this study is relatively small, which can be considered a limitation of the work. We suggest that because of the learning curve, the improvement in arc ROM is limited. All surgical interventions were made by the same surgeon (KE) using a standard surgical procedure, and the study also featured a long-term follow-up period, and these factors increase the strength of the present study. Various complications, including neurovascular injuries, may be encountered while opening the arthroscopic portals, as reported by Kim et al. [24], in a case series of 63 patients. The surgery should be carried out by the same surgeon who completed the learning curve to avoid such complications. Indeed, no postoperative neurovascular complications were witnessed in our patients, and we believe that the very careful handling during the opening of the portals and the fact that the surgical procedure was performed by the same experienced surgeon in elbow arthroscopy, contribute to this outcome.

The long follow-up period, and the fact that the examination of the patients in the final follow-up was done by a physician other than the surgeon who performed the procedures, and that the statistical analysis was made by a different specialist, increases further the strength of this study.

## Conclusions

Elbow arthroscopy is an important tool in the diagnosis and management of injuries to the elbow joint. Loss of motion is a common complication after elbow trauma and can significantly interfere with the ability to perform activities in daily life. Elbow contractures commonly result from both intrinsic and extrinsic factors, causing limited motion. Nonsurgical treatment, including physiotherapy and static splinting, can restore a functional arc of motion in some patients. The diagnosis and a sound understanding of related pathology are crucial to ensure a successful procedure. The arthroscopic release and debridement of related structures is an effective and safe option for improving the ROM of the joint in patients with stiff elbow, whether in post-traumatic or non-traumatic cases.
